# Supplementary material for: Focal Cortical Dysplasia Type Ⅲ Related Medically Refractory Epilepsy: MRI Findings and Potential Predictors of Surgery Outcome
Source: Diagnostics (Basel). 2021 Nov 29;11(12):2225. doi: 10.3390/diagnostics11122225 (PMC8699898; doi:10.3390/diagnostics11122225)
Supplement: Supplementary file 1 [file diagnostics-11-02225-s001.zip › diagnostics-1426225-supplementary.pdf]

Table S1: MRI protocol

sequence	TR (ms)	TE (ms)	FOV (mm^2)	ST (mm)	slice gap (mm)
T1WI	678	15	220 × 209	5	1
T2WI	5728	110	220 × 187	5	1
T2W FLAIR	9480	120	220 × 188	5	1
